# Pericecal Hernia Presenting as Acute Appendicitis

**DOI:** 10.7759/cureus.6868

**Published:** 2020-02-04

**Authors:** Anupam K Gupta, Michael P Kucharik, Arye Lavin, Miguel Lopez-Viego

**Affiliations:** 1 Surgery, Charles E. Schmidt College of Medicine, Florida Atlantic University, Boca Raton, USA; 2 Internal Medicine, Charles E. Schmidt College of Medicine, Florida Atlantic University, Boca Raton, USA; 3 General & Vascular Surgery, Bethesda Hospital East, Boynton Beach, USA

**Keywords:** hernia, pericecal, internal hernia, acute appendicitis, paracecal hernia, pericecal hernia

## Abstract

We would like to report an unusual case of a pericecal hernia in a 93-year-old female. The patient did not report a history of previous abdominal surgery and presented with acute abdominal pain, constipation, nausea, and vomiting. Diagnosis was made with computerized tomography and laparoscopy was performed, which was significant for loops of small bowel in the pericecal region in a defect of peritoneum. The small bowel loops were mobilized back in the intraperitoneal location and the defect was closed to prevent further herniation. Our case is an extremely rare presentation of a rare condition, as there have only been five reported cases of pericecal hernias that required immediate surgical intervention and outlined explicit details of the surgical procedure. Pericecal hernias are unusual occurrences and occur more frequently in older women. Since the clinical signs and symptoms mimic acute appendicitis, delays in diagnosis are common. Thus, this case highlights the importance of suspecting strangulated internal hernias in patients with signs and symptoms of acute appendicitis to prevent significant morbidity and mortality.

## Introduction

Hernias can be broadly categorized as internal and external. An internal hernia is the protrusion of visceral contents through a congenital or acquired opening within the peritoneal cavity, whereas external hernias occur through an opening in the abdominal wall [1]. Internal hernias comprise less than 1% of all hernias, but constitute up to 5.8% of all cases of small bowel obstructions [2]. If left untreated, internal hernias are associated with a mortality rate that exceeds 50%. Among the subsets of internal hernias, pericecal hernias comprise approximately 13% of all cases [2]. Early diagnosis of this rare condition can aid in laparoscopic management and prevent significant morbidity and mortality [3].

Both hernias and appendicitis can present with abdominal pain. The pain associated with appendicitis is classically initially dull, migratory, and periumbilical, but becomes sharp in the right lower quadrant once the parietal peritoneum is irritated by a distended and inflamed appendix. With a lifetime risk of 8.6% in males and 6.7% in females, appendicitis is a common pathology that can mask infrequent causes of right lower quadrant abdominal pain, such as a strangulated pericecal hernia [4].

## Case presentation

We would like to report a 93-year-old female with no previous abdominal surgery, presenting with a three-day history of constipation and one-day history of abdominal pain, nausea and vomiting. The abdominal pain was described as sharp and located in the right lower quadrant. On presentation, the patient was afebrile and did not complain of recent chills, night sweats, melena, weight loss, or previously altered bowel habits. Her past medical history was significant for hyperlipidemia and gastroesophageal reflux disease. She had no previous surgeries and her medication list included simvastatin and ranitidine. The patient’s vital signs were notable for a temperature of 99.4°F, heart rate of 98 bpm, blood pressure 110/90 mm Hg, respiratory rate of 18 breaths per min, and an oxygen saturation of more than 95% on room air. Clinically, abdomen was soft, but distended with tenderness on palpation in the right lower quadrant with sluggish bowel sounds.

At this point, due to the patient's history and nature of the abdominal pain, our leading diagnosis was acute appendicitis followed by small bowel obstruction with a possible corresponding hernia. Since the patient had a negative past abdominal surgical history, adhesions would be an unlikely cause of small bowel obstruction. Other causes of right lower quadrant abdominal pain such as nephrolithiasis and ischemic bowel were less likely due to the absence of hematuria or hematochezia/melena.

**Figure 1:**
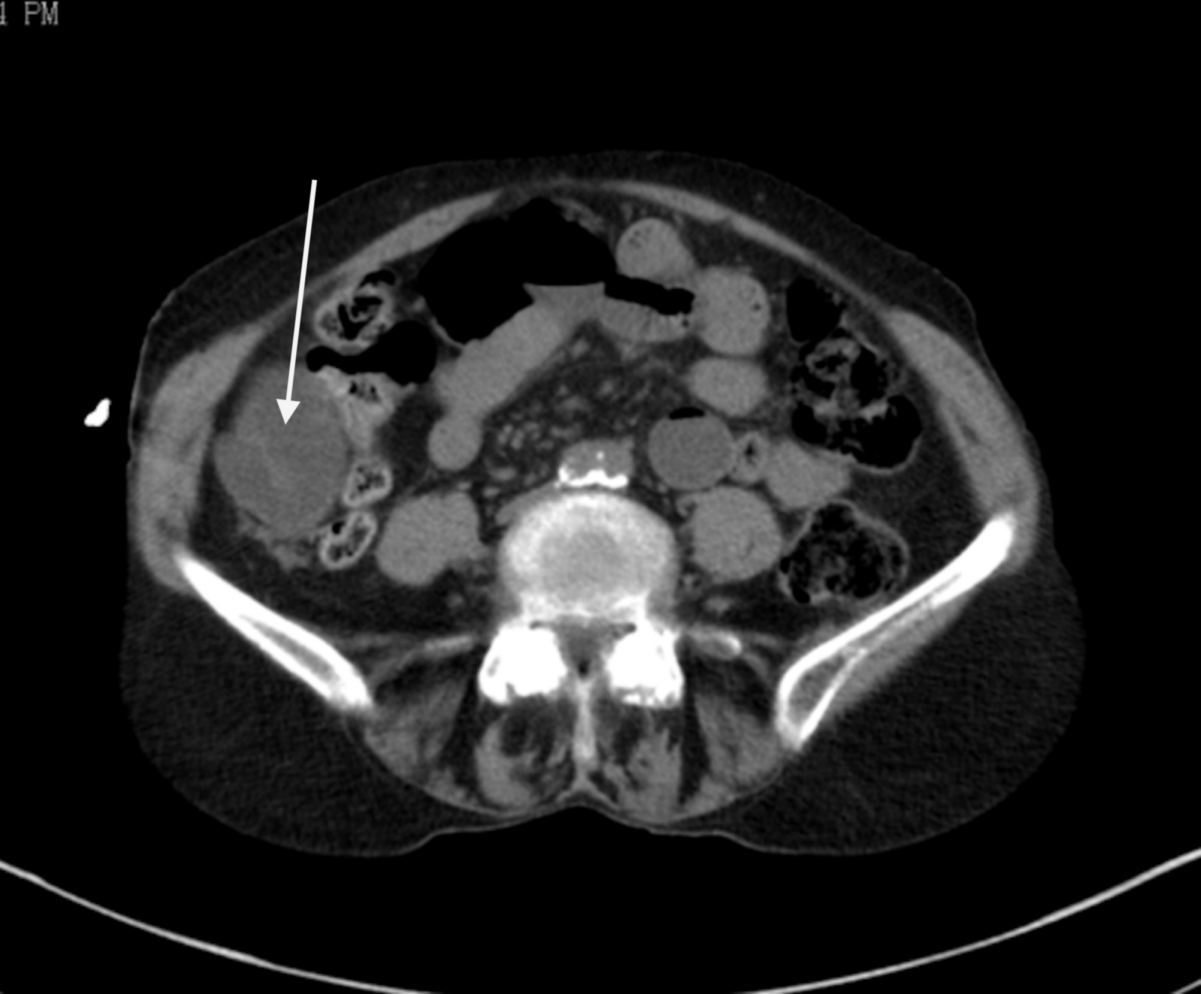
Dilated small bowel loop lateral to cecum in the right paracolic gutter.

**Figure 2:**
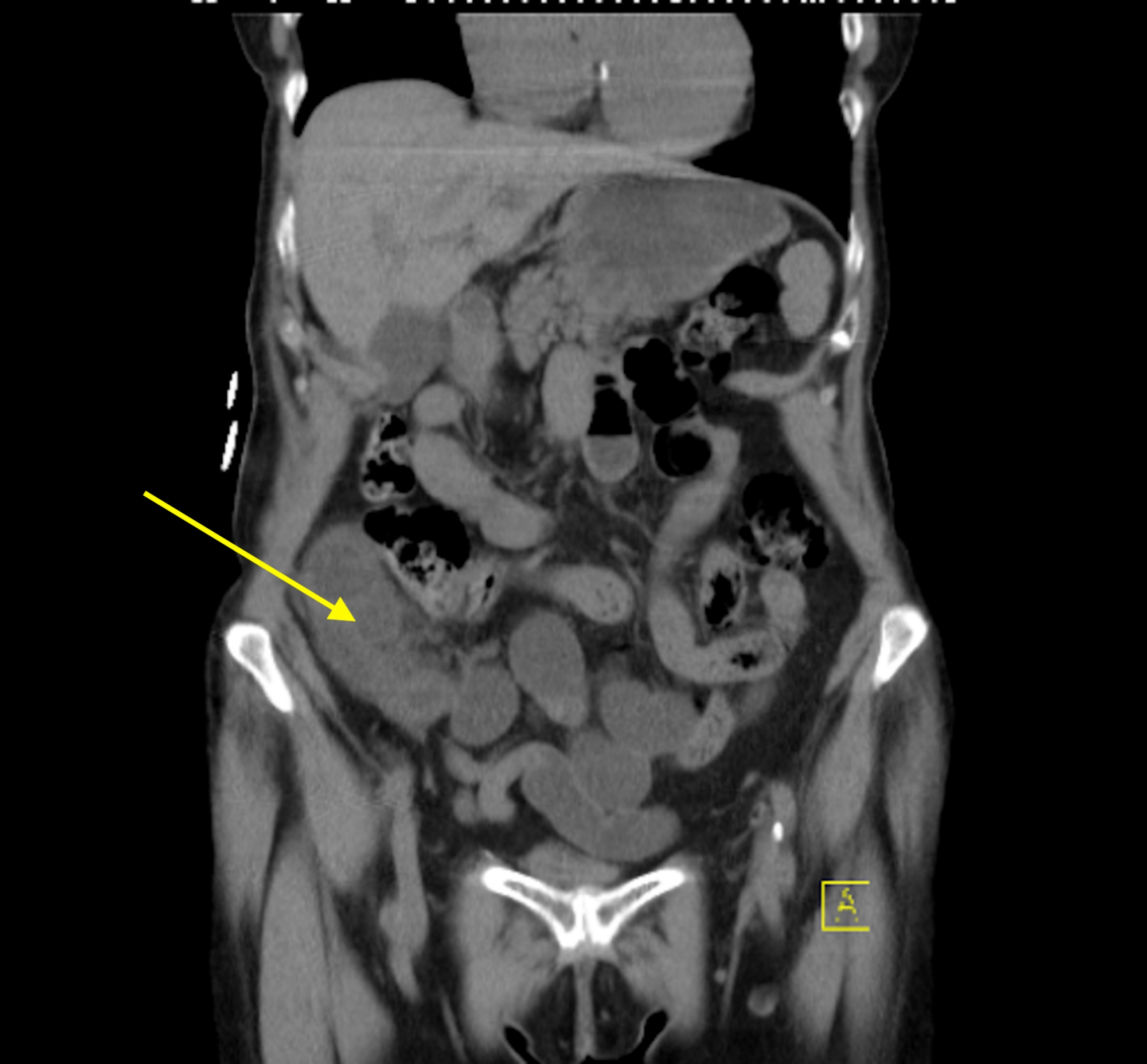
Small bowel loop lateral to cecum.

Her routine blood work was significant for an elevated white count to 12,000/mL with 89% neutrophilia, hemoglobin 12.9 g/dL, hematocrit of 30%, platelet count of 150,000/mL and basic electrolyte profile showed sodium of 140 mEq/mL, potassium of 3.7 mEq/mL, blood urea nitrogen of 37 mg/dL, creatinine of 1.1 mg/dL, and C-reactive protein of 5.5 mg/L. A noncontrast computed tomography (CT) scan of the abdomen and pelvis was performed to further investigate. This revealed small-bowel loops in the right lower quadrant lateral to the ascending colon. The loop of bowel appeared distended and was associated with some mesenteric fat stranding (Figures 1, 2).

After fluid resuscitation and bowel rest, the patient was considered for surgery as she continued to complain of increasing pain in right lower quadrant, which was not relieved with ketorolac.

**Figure 3:**
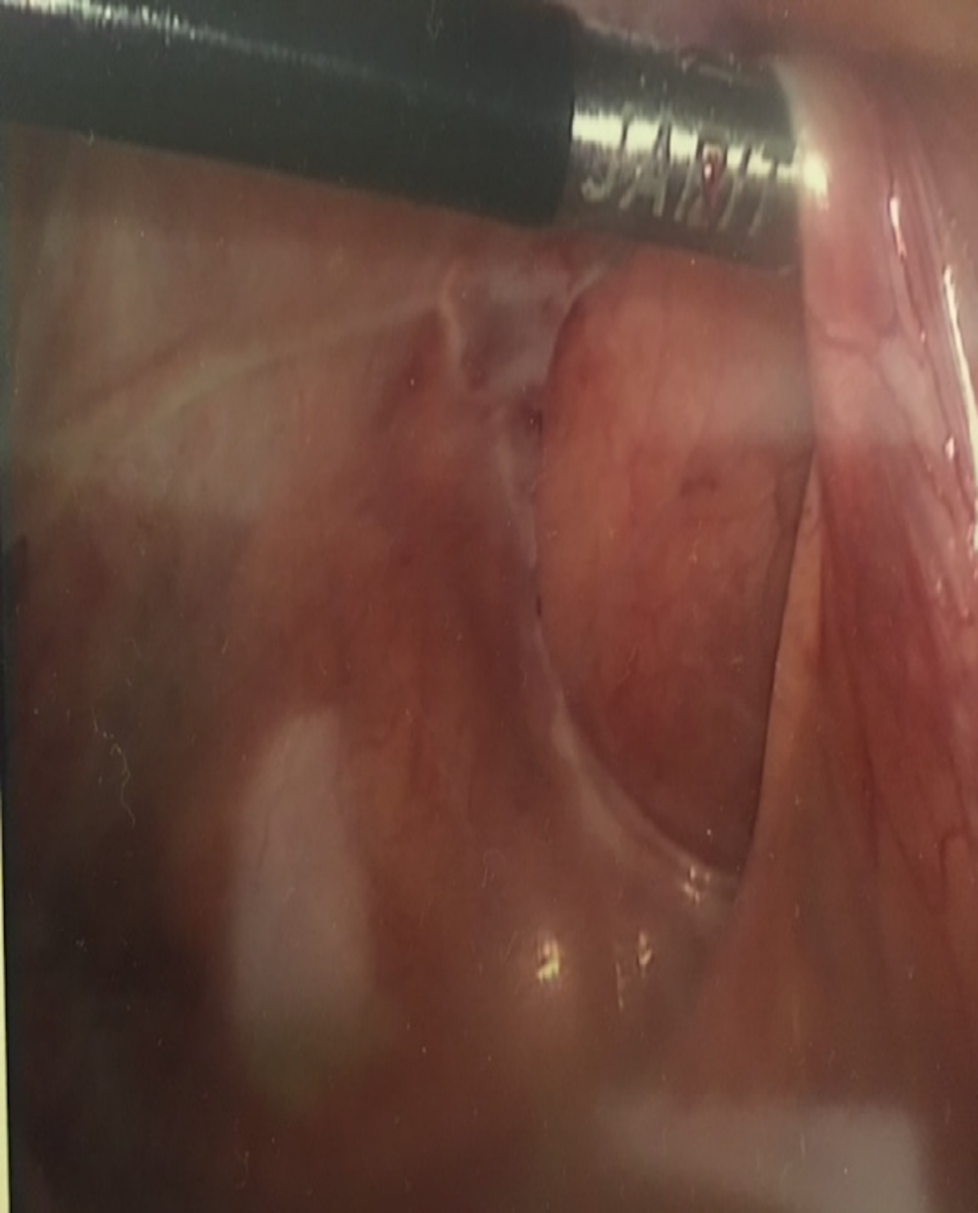
Intraoperative picture showing the pericecal defect which was subsequently closed with vicryl tackers.

The patient was taken to the operating room and a diagnostic laparoscopy was performed using a 10-mm video port at the umbilicus. An additional midline 5-mm port was placed in the suprapubic region. Loops of small bowel were visualized in the pericecal region in a defect of peritoneum. The small bowel loops were mobilized back in the intraperitoneal location using a grasper and blunt dissection. The peritoneal defect was identified (Figure 3) and this natural orifice was subsequently overlapped with the surrounding peritoneum and closed with vicryl tackers to prevent further herniation. The bowel loops appeared congested and edematous, however, were viable in nature so no resection anastomosis was performed.

The patient withstood the procedure well and had a normal postoperative course. The patient was tolerating diet and ambulatory on postoperative day 1. On postoperative day 2, the patient was subsequently discharged with an in-office follow-up scheduled for postoperative day 7. On postoperative day 7, the patient's surgical incisions were dry, clean, and intact. Her bowel habits returned to normal and she denied nausea, abdominal pain, fever, chills, melena, hematochezia, or abdominal pain.

## Discussion

Internal hernias can result from natural orifices or those made from procedures, such as Roux-en-Y gastric bypass [2]. Most common natural orifices include paraduodenal (53%), pericecal (13%), foramen of Winslow (8%), transmesenteric (8%), and intersigmoid (6%) [3, 5]. Pericecal hernias are uncommon and mimic acute appendicitis in initial stages with pain and nausea followed by small bowel obstruction in the later stages [2]. The pericecal recesses are formed around the fifth month of gestation when the bowel loops return to the abdominal cavity [3]. There are four defined pericecal recesses: superior ileocecal, inferior ileocecal, retrocecal, and paracolic sulci [2]. When obstructed, patients complain of obstipation and right lower quadrant tenderness [5].

Radiograph imaging, specifically non-contrast CT scans, reveals loops of bowel, usually posterolateral to the cecum and right paracolic gutter. Also present can be signs of small bowel obstruction, such as air fluid levels, dilated loops of small bowel, and a transition point [3]. Early diagnosis and use of laparoscopy can help reduce the hernia and minimize morbidity.

A literature review on PubMed including the terms “pericecal hernia” and “paracecal hernia” yielded 27 and 24 matches, respectively. Articles that did not provide surgical details or utilize urgent surgical intervention were excluded from our literature review. One manuscript reported three cases of pericecal hernia in a series of 533 cases of small bowel obstruction [6]. Among the remaining 13 cases, the demographics included a mean age of 69 years, with eight female and five male patients (Table 1). Only three patients had prior surgical history, two of which underwent appendectomy near the pericecal region. Eight of these patients were managed by exploratory laparotomy, while five were managed laparoscopically (Table 1) [7-17].

**Table 1:** Published cases of pericecal hernias treated with urgent surgical intervention.

Serial No.	Age/Sex	Past surgical history	Surgery	Source
1	88/F	Unknown	Exploratory laparotomy	(Kumar et al., 2015 [15])
2	92/M	Cholecystectomy	Laparoscopic	(Ogami et al., 2016 [3])
3	65/M	None	Laparoscopic	(Sasaki et al., 2016 [16])
4	34/M	None	Exploratory laparotomy	(Kleyman et al., 2013 [10])
5	69/M	None	Exploratory laparotomy	(Lu et al., 2017 [11])
6	67/F	Appendectomy	Exploratory laparotomy	(Lu et al., 2017 [11])
7	70/F	None	Exploratory laparotomy	(Nishi et al., 2011 [13])
8	84/F	None	Exploratory laparotomy	(Jang et al., 2011 [8])
9	63/M	Unknown	Exploratory laparotomy	(Shibuya et al., 2010 [17])
10	43/F	Unknown	Laparoscopic	(Kabashima et al., 2010 [9])
11	74/F	Laparoscopic appendectomy	Laparoscopic to minilaparotomy	(Hirokawa et al., 2007 [7])
12	59/F	Unknown	Exploratory laparotomy	(Molto Aguado et al., 2007 [12])
13	90/F	Unknown	Laparoscopic	(Omori et al., 2003 [14])

When comparing the aforementioned cases with our case, our patient’s presentation is particularly notable because of her age, lack of past surgical history, and surgical method. According to our literature review, there were just two other nonagenarian patients that suffered from this condition [3,13]. Moreover, our patient was the only nonagenarian patient to present with a negative past surgical history and without risk factors for hernia including pregnancy, recent weight lifting, constipation, or weight gain.

Additionally, immediate diagnostic imaging allowed our patient to be managed with minimally invasive laparoscopic surgery. Based on our literature review, the nonspecific signs and symptoms of strangulated pericecal hernias delayed diagnostic imaging, which ultimately resulted in an exploratory laparotomy in a majority of cases. Thus, our case can be utilized as a guide for the early diagnosis and management of patients with potential strangulated internal hernias.

## Conclusions

Pericecal hernia is an unusual occurrence and is seen to occur more frequently in older women. The clinical signs and symptoms often mimic acute appendicitis, which typically causes a delay in diagnosis. Clinical findings are often insufficient to make a diagnosis. Early diagnosis can be made with imaging like computed tomography. In patients, early laparoscopy can help confirm diagnosis and reduce the internal hernia. The internal hernial orifice should be closed or enlarged to prevent further herniation. Delay in diagnosis can result in hernia getting complicated needing an exploratory laparotomy and prolonged morbidity.
